# *In Trans* Complementation of Lethal Factor Reveal Roles in Colonization and Dissemination in a Murine Mouse Model

**DOI:** 10.1371/journal.pone.0095950

**Published:** 2014-04-24

**Authors:** David E. Lowe, Jason Ya, Ian J. Glomski

**Affiliations:** Department of Microbiology, Immunology, and Cancer Biology, University of Virginia, Charlottesville, Virginia, United States of America; East Carolina University School of Medicine, United States of America

## Abstract

Lethal factor (LF) is a component of the *B. anthracis* exotoxin and critical for pathogenesis. The roles of LF in early anthrax pathogenesis, such as colonization and dissemination from the initial site of infection, are poorly understood. In mice models of infection, LF-deficient strains either have altered dissemination patterns or do not colonize, precluding analysis of the role of LF in colonization and dissemination from the portal of entry. Previous reports indicate rabbit and guinea pig models infected with LF-deficient strains have decreased virulence, yet the inability to use bioluminescent imaging techniques to track *B. anthracis* growth and dissemination in these hosts makes analysis of early pathogenesis challenging. In this study, the roles of LF early in infection were analyzed using bioluminescent signature tagged libraries of *B. anthracis* with varying ratios of LF-producing and LF-deficient clones. Populations where all clones produced LF and populations where only 40% of clones produce LF were equally virulent. The 40% LF-producing clones *trans* complimented the LF mutants and permitted them to colonize and disseminate. Decreases of the LF producing strains to 10% or 0.3% of the population led to increased host survival and decreased *trans* complementation of the LF mutants. A library with 10% LF producing clones could replicate and disseminate, but fewer clones disseminated and the mutant clones were less competitive than wild type. The inoculum with 0.3% LF producing clones could not colonize the host. This strongly suggests that between 10% and 0.3% of the population must produce LF in order to colonize. In total, these findings suggest that a threshold of LF must be produced in order for colonization and dissemination to occur *in vivo*. These observations suggest that LF has a major role in the early stages of colonization and dissemination.

## Introduction

*Bacillus anthracis* is a Gram-positive organism that is the etiological agent of anthrax [1]. Anthrax can occur via cutaneous, inhalational, and gastrointestinal routes; the latter two forms are highly lethal in livestock and humans [2]. The infectious particle for *B. anthracis* is the spore, which is characterized as metabolically inert and highly resistant to many forms of bactericidal killing [3]. Once inside the host, the spore senses the host environment and germinates to form the vegetative bacillus [4]. After germination, the bacilli are able to multiply to high numbers in the host due to their potent exotoxin and anti-phagocytic capsules [1]. Eventually, the bacteria cause the death of the host, inducing the bacilli to undergo sporulation and subsequently releasing spores into the soil as the tissue decays.

*B. anthracis*' exotoxin can intoxicate many types of cells, which leads to profound changes in the host physiology and promotes disease [5]. The exotoxin is composed of the host-cell binding component, protective antigen (PA), and two enzymatic subunits, lethal factor (LF) and edema factor (EF) [6]–[8]. PA can bind two host exotoxin receptors, capillary morphogenesis protein-2 (CMG2 or ANTXR2) and tumor endothelial marker 8 (TEM8 or ANTXR1) [9], [10]. Either in the serum or after binding with the receptor, PA is cleaved by a protease which allows PA to oligomerize [11]–[14]. After PA oligomerization, LF and/or EF can bind to the PA-receptor complex, which leads to internalization into the cell by clatherin-mediated endocytosis [15]. When LF has bound to the PA oligomer, it is referred to as lethal toxin (LT). Similarly, when EF has bound to PA, it is referred to as edema toxin (ET). Once internalized by the cell, a decrease in pH in the endocytic vacuole causes PA to form a pore that allows LF and/or EF to translocate to the cytoplasm [12], [16]–[18]. Once in the cytoplasm, LF cleaves most mitogen-activated protein/extracellular signal-regulated kinase kinases (MEKs) and EF increases cAMP levels in the cell [19], [20]. Broadly, the exotoxin has two roles: allow the bacteria to proliferate to high numbers and induce host death by targeting key host tissues [21], [22]. However, what roles these exotoxin components are playing early in infection and which roles are necessary for bacterial dissemination from the initial site of infection remain to be clarified.

The exotoxin is rapidly expressed in the host after germination and the exotoxin production is critical for both the establishment of the infection and death in most animal models [22]–[25]. Both LF and EF are capable of increasing endothelial permeability, reducing chemotaxis, limiting the bacteriocidal activity of immune cells, and inducing cell death [5], [8]. Deletion of CMG2/ANTXR2 in myeloid cells led to host resistance to infection with an uncapsulated strain in mice [26]. LF in particular is important for disabling the immune response early in infection [26], [27]. In addition to targeting local immune cells at the initial site of infection, the exotoxin affects key tissues which contribute to the death of the animal [21]. Yet, the amount of LF necessary for either establishment or dissemination is not well understood. Further, it is not known if decreased amounts of LF lead to reductions in bacterial burden or delays in dissemination.

The individual roles of LF and EF *in vivo* have been examined using genetic deletions to delineate their roles in pathogenesis. Most animal models require PA to be secreted for infection to occur, underscoring the importance of the exotoxin in disease [22], [23], [28]. In some animal models, such as rabbits, the presence of either LF or EF in addition to PA is necessary for disease [22], [28]. Inhalational and subcutaneous rabbit infections with either the LF-deficient mutant (ΔLF) or the edema factor mutant (ΔEF) strains led to higher 50% lethal doses and increased host survival compared to infection with wild type *B. anthracis* [22], [25], [28]. Interestingly, the secreted exotoxin could not be complemented *in trans* when rabbits were infected with a mixed inoculum of capsulated fully toxigenic *B. anthracis* and a capsulated ΔPA mutant [22]. This suggested that the protective effect of the exotoxin is limited to an area near the bacilli (*i.e*. each bacterium must secrete its own exotoxin in order for the exotoxin to successfully protect that single bacterium). Additionally, it was concluded that the primary role of the exotoxin was to disable the immune response in the early stages of infection to allow for dissemination [22].

It is also difficult to analyze the early stages of infection in most animal models due to the asymptomatic and asynchronous nature of anthrax infections [23]. To overcome these challenges, mouse models of anthrax used bioluminescent strains of *B. anthracis*; this allows for real time analysis of dissemination and a deeper understanding of the early events. In mouse models, studies investigating the effect of the individual exotoxin components using bioluminescent strains have shown differing results that are dependent on the presence of capsule. Unlike other animal models, mice can be infected with non-toxigenic, capsulated strains and will have the disease progress to death [29]. In capsulated *B. anthracis* strains, the absence of LF led to an altered dissemination pattern compared to ΔEF or the parental strains in inhalational infections [30]. In mice infections with the uncapsulated *B. anthracis* strain, ΔLF, ΔPA, and ΔLF/ΔEF/ΔPA strains could not disseminate while ΔEF strains had decreased virulence [23]. While mouse models have differences from other animal models, they allow for real time analysis of the early stages of anthrax infection that are otherwise difficult to study. Therefore, the mouse model of infection was used in these studies to further understand the role of LF in early pathogenesis.

While previous studies have indicated that the exotoxin is important for disease at the initial sites of colonization, the precise roles of the exotoxin for colonization and dissemination are not well defined. Thus, investigations are needed in order to better understand the mechanisms of dissemination from the portals of entry and to better define a rational therapeutic window for potential exotoxin-targeting interventions. Although there have been advances in quantifying the amount of exotoxin present within the host and the amount needed to alter the host immune response, it is not well understood how much exotoxin is needed for dissemination to occur in animal models [27]. By varying the amount of LF produced by a population, it is possible to examine the effects of the LF concentration on the bacteria's ability to thrive and spread the infection. Moreover, this data would better inform researchers on the efficacy of therapeutics during infection. If a tiny amount of exotoxin components are needed, therapeutics targeting the exotoxin would be most successful at early stages of disease and should be used as prophylaxis before the infection initiates. Whereas if a larger amount of exotoxin is needed in order for the disease to progress, it would suggest that dissemination and disease relies heavily on the exotoxin. If such a scenario occurred, anti-toxin therapeutics could be used at a later stage of disease to prevent morbidity.

To better understand the roles of LF in the early stages infection, mice were challenged with *B. anthracis* libraries that produced lower amounts of LF as a population. Signature tag libraries were made with varying ratios of LF expressing (LF^+^) and ΔLF mutants. The libraries had 100%, 40%, 10% or 0.3% of the population comprised of LF-producing strains; the remainder of the population was comprised of ΔLF mutants. The bacteria in these libraries had unique DNA tags inserted into the chromosome to further delineate the role of LF on the disseminating population structure. When the bacteria were induced *in vitro* to produce exotoxin with R media, there was no difference in the ability of the 100% LF^+^ or 40% LF^+^ inoculum to lyse macrophages. However, the 10% and 0.3% LF^+^ inoculums were not able to induce significantly more killing than the media control. Similarly, *in vivo* infections of mice found no difference in survival between mice infected with the 100% LF^+^ and 40% LF^+^ libraries. Mice infected with the 40% LF^+^ library also had no difference in dissemination kinetics and or bacterial burden when compared to the 100% LF^+^ inoculum. However, mice infected with the 10% and 0.3% LF^+^ library demonstrated increased survival compared to the 100% LF^+^ library. Infection with a 0.3% LF^+^ inoculum never colonized, disseminated, or caused mortality; however, the 10% LF^+^ library could colonize the ear for several days but was less capable of dissemination. Since it was previously shown that the protective role of the exotoxin cannot be complemented *in trans*, it was hypothesized that the ΔLF clones would not survive at the initial site of infection and the population would not be able to disseminate. However, when mice were infected with the 40% and 10% LF^+^ inoculum, the ΔLF bacteria were still capable of survival and dissemination. In contrast, infections with 10% LF^+^ inoculum demonstrated decreased rates of dissemination and were less competitive than their LF^+^ counterparts compared to the 100% LF^+^ inoculation. These data suggest that discrete amounts of LF are necessary for colonization and dissemination in a mouse model. Furthermore, secreted LF can act *in trans* to promote the growth of ΔLF bacteria when at near equal numbers of wild-type and mutant bacteria in a subcutaneous mouse model.

## Materials and Method

### Ethics statement for animal model studies

All studies with mice were performed under the principles described in the Guide for the Care and Use of Laboratory Animals of the National Institutes of Health [31]. The techniques as well as husbandry of these animals were approved by the University of Virginia Animal Care and Use committee (protocol #3671) and designed to minimize distress and pain for the animals.

### Bacterial strains and culturing

*Bacillus anthracis* 7702 Sterne strain (pX01^+^, pX02^−^) was kindly provided by Molly Hughes, Division of Infectious Diseases, University of Virginia (Charlottesville, VA). ΔLF 7702 was a kind gift from Scott Stibitz [32]. Construction of LF^+^ strains with recombinant DNA tags has been previously described and the same cloning and allelic exchange strategy was used to insert recombinant tags into ΔLF strains [33]. Previous studies have demonstrated that these clones had no defect in *in vitro* growth rates or ability to disseminate *in vivo*. Strains were made luminescent by transforming pIG6-19 vector into either the 7702 or ΔLF 7702 strain and selecting for erythromycin (Erm) resistance. Briefly, pIG6-19 is a suicide vector that inserts the plasmid into the pX01 plasmid through a single crossover event and does not interrupt the gene encoding protective antigen or its native promoter. The pIG6-19 plasmid contains the *luxABCDE* operon under the control of the protective antigen promoter allowing for luminescence *in vivo* and was a kind gift from Michèle Mock [24]. The strain BIG23L is a luminescent derivative of ΔLF 7702 and does not contain any insertion in the *eag* locus. DNA tagged ΔLF and LF^+^ strains were also transformed with pIG6-19 to allow for bioluminescent analysis. DNA tag sizes were confirmed by PCR analysis and bioluminescence was confirmed by incubating bacteria overnight on Cap media using the IVIS100 (Perkin Elmer, MA) [34]. Clones were then induced to sporulate by plating bacteria on NBY media with 5 µg/mL Erm at 30°C for 10 days. The spores were then collected from plates and purified on an Omnipaq gradient (350 mg of iodine/mL, GE Healthcare, NJ) as described previously [24], [35], [36]. Spores of DNA tagged clones were CFU enumerated and mixed to make libraries with various ratios of LF^+^ and ΔLF bacilli. These libraries were kept at 4°C or kept on ice until used for experiments.

### Cell culture and cytotoxicity measurements

The libraries were induced to produce exotoxin *in vitro* by incubating in R media [37]. Libraries were grown from spores in LB supplemented with 5 µg/mL Erm and shaken overnight at 37°C. The overnight cultures were then diluted 1∶10 in 100 µL LB in a 96 well plate in a humid chamber and incubated overnight at 37°C overnight without shaking. Finally, fresh R media was prepared and supplemented with 0.8% Sodium bicarbonate and the overnight bacterial cultures were diluted 1∶10 into the R media in a 96 well plate. Samples were incubated in a 37°C incubator with 5% CO_2_ without shaking for 6 hours. The supernatant was collected and spun down to remove any bacteria. Supernatants were kept at −80°C until needed for cytotoxic experiments.

RAW264.7 cells (ATCC, VA) were grown in high glucose DMEM (Invitrogen-Gibco, CA) supplemented with 10% fetal bovine serum (Invitrogen-Gibco, CA) and 1000 units/mL penicillin-streptomycin (Invitrogen-Gibco, CA) and incubated at 37°C with 5% CO_2_. For cytotoxicity assays, 50,000 cells in 200 µL were seeded into a 96 well plate and allowed to adhere for at least 2 hours. The supernatants or R media were added at 1/10^th^ the final volume and allowed to incubate for 4 hours. In some wells recombinant LF was added at a final concentration of 2.5 µg/mL (27.8 nM) in 200 µL. Cytotoxicity was measured by %LDH released into the media using the Promega CytoTox96 Non-radioactive Cytotoxicity (Promega). The %LDH release was normalized to the amount of LDH released by the supernatant from the 100% LF^+^ inoculum.

### Quantification of the amount of LF produced by the various LF-producing libraries

Relative amounts of LF were quantified using previously described methods [33]. Briefly, the exotoxin-containing supernatants used in the cytotoxicity assays were quantified using the D_C_ Protein Assay Kit (Bio-Rad, Hercules CA), normalized to 40 µg in a final volume of 30 µL of 1x Laemmli buffer, and heated at 95°C for 4 minutes. Total protein amounts were measured by staining with MemCode Reversible Protein stain (Thermo Scientific, Rockford IL) and relative amounts quantified by densitometry using the ChemiDoc XRS Imaging system and Image lab v4.0 (Bio-Rad, Hercules CA). The membrane was blocked in a 4% Non-fat milk/TBS +0.05% Tween-20 (TBS-T) solution at 4°C overnight and then incubated for 1 hour with 1∶2500 α-LF (DD-6; BEI, Manassas, VA). After washing with TBS-T, the membranes were incubated for 1 hour with 1∶1000 goat α-mouse IgG Alkaline phosphatase. Bands were developed by incubating with Immuno BCIP/NBT substrate solution (MP Biomedicals, Solon OH) until bands were clearly visible. The amount of LF was then quantified using densitometry via the ChemiDoc XRS Imaging system and Image lab v4.0 (Bio-Rad, Hercules CA).

Relative quantitation was calculated using the following formula:
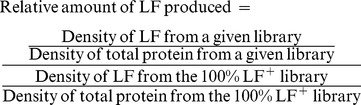



### Mouse infection and bioluminescent monitoring of dissemination

All mice were 6–12 week A/J mice (Jackson Labs) and were housed in pathogen-free caging at the University of Virginia. Mice were infected by subcutaneous injection in the ears as previously described [24], [36]. Briefly, mice were anesthetized with a 3% Isoflurane-oxygen (v/v%) mix using a Isotec 5 vaporizer (Absolute Anesthesia) and 10 µL of spores in PBS were injected into the ear using a 0.5 cc syringe. The inoculum varied between 2×10^5^ and 5×10^5^ spore CFU per mouse. Mice were observed once a day for 7 days after infection and bioluminescence was monitored using an IVIS100 (Perkin Elmer). The bioluminescent radiance was measured using LivingImage Software (version 3.2, Perkin Elmer). For fold difference of luminescent radiance over background, a region of interest (ROI) was measured over the luminescent ear and an ROI of the same size was measured over the uninfected ear. Fold difference was calculated as the radiance of the infected ear divided by the radiance on the non-infected ear. Any mouse with luminescence detected in the kidneys was considered to have a fatal infection and was euthanized to reduce pain or distress. Bacterial burden in tissues were determined as previously described [33]. The percent germination that occurred in tissues was determined by comparing the CFU enumerations from heated and unheated tissue homogenates. The heated portion of the tissue was incubated at 65°C for 20 minutes, killing all vegetative bacilli in the homogenates and leaving the heat resistant spores intact. Whereas the unheated portion of the tissue homogenate contained both bacilli and spores. The percent germination can then be calculated by: (1- (CFU per organ of the heated population/CFU per organ of the unheated population))*100.

### Clonal analysis to determine proportions of signature tagged mutants in organs

The proportion of clones in the organs were determined by selecting 48 clones from each organ of a mouse that had a disseminated infection and using PCR to determine the size of the signature tag. Since small numbers of clones could skew the clonal analysis, organs were only analyzed if there were >300 CFU per organ. Colonies were chosen at random and lysed using the HotSHoT method to extract DNA [38]. Each clone had a tag inserted into the *eag* locus and PCR amplification used primers which flanked the insertion region and conditions as previously described [33].

### Statistical analysis

Statistical analysis for Student's T Test, one way ANOVAs, two way ANOVAs, and post-hoc tests were calculated using Graphpad Prism (version 6.03, Graphpad Software). Fisher exact tests were calculated using R: A language and environment for statistical computing [39].

## Results

### Reductions in LF-producing bacilli lead to increases in host survival

The elimination of LF production decreases virulence in most animal models and is required for inhalational infections to occur in the Sterne-A/J model [22], [23], [28]. Few studies, however, have analyzed the relative amount of LF that is required for colonization or dissemination from the initial site of the infection. *B. anthracis* strains are highly conserved on a genomic level and strains found in nature have not been reported to lack or express lower levels lethal factor. Therefore, to assess the role of LF for dissemination from the portal of entry, several signature tag libraries were engineered with decreasing ratios of 7702 (LF^+^) to Δ*lef* 7702 (ΔLF) to experimentally address the effects of lower levels of LF production. The amount of LF that could be produced by a population was controlled by altering the proportion of the inoculum that encodes for LF. While techniques exist that can quantify the amount of LF within a host, difficulties still exist for quantifying the amount of LF that has been delivered to the cells and thus are inducing a biological effect [27], [36], [40]. By adjusting the amount of LF produced by a population, one can determine the effect of this amount on dissemination without making assumptions based on exotoxin internalization rates or clearance from the host. Additionally, the amount of PA and EF produced by the library would remain the same as LF decreased. A previously developed bioluminescent signature tag library, which differed only by the presence of uniquely sized pieces of recombinant DNA, was constructed in the ΔLF and LF^+^ 7702 strains [33]. The ΔLF clones either had no DNA tag inserted, designated BIG23L, or either a 300 bp or 400 bp DNA tag inserted in the S-layer *eag* locus in the chromosome. The LF^+^ clones had either a 500 bp or a 700 bp DNA tag inserted into the *eag* locus. Previous work established that insertion of the tag in these clones did not alter the clones' growth *in vitro* or ability to disseminate *in vivo* [33]. The proportions in each inoculum library were constructed to have 3 ΔLF clone to 2 LF^+^ clones (40% LF^+^), 9 ΔLF to 1 LF^+^ clone (10% LF^+^), or 299 ΔLF to 1 LF^+^ clone (0.3% LF^+^).

**Figure 1:**
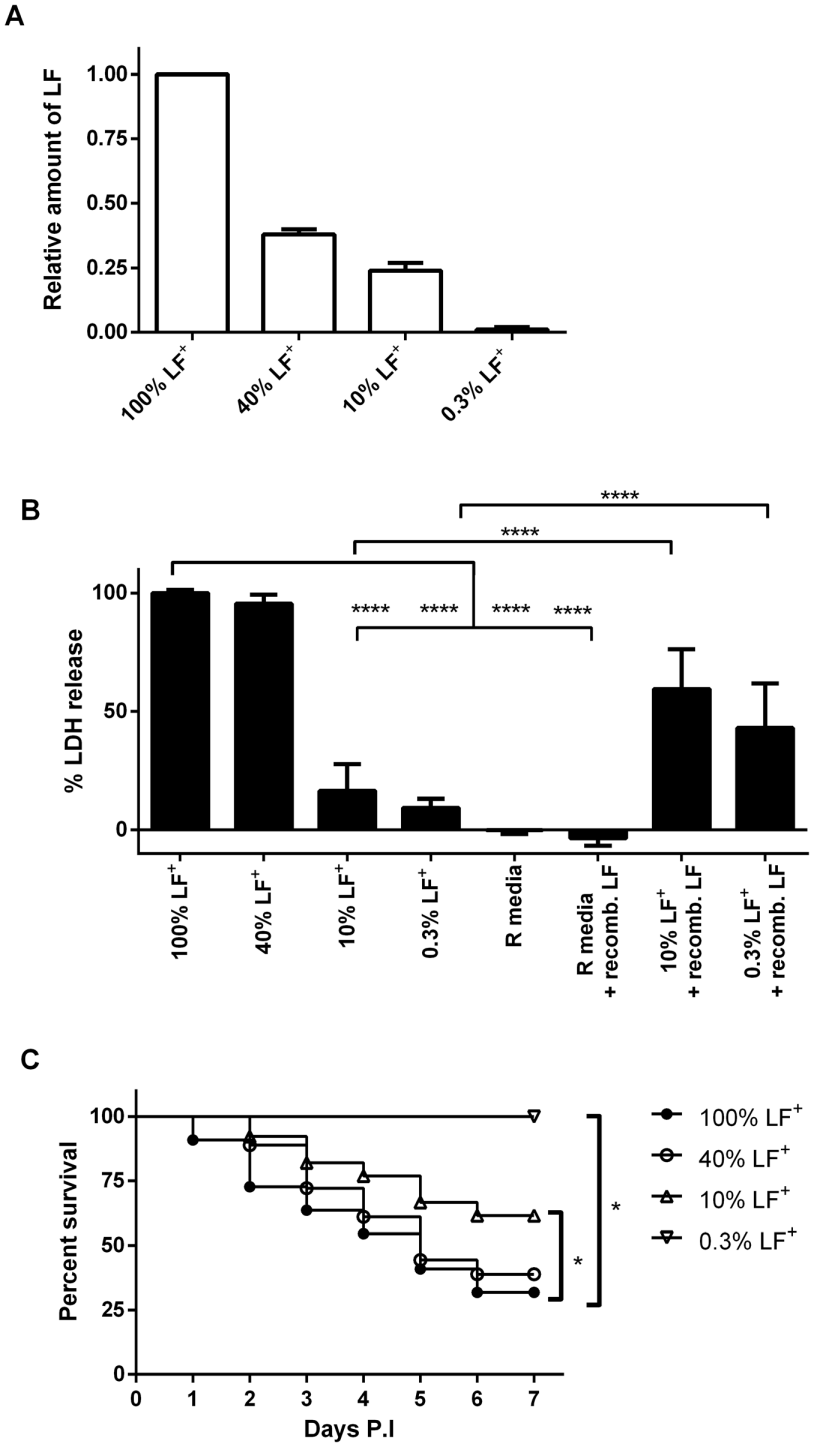
Decreasing LF producing clones in a signature tag library led to reductions in virulence. **A**) Decreasing the amount of LF-producing clones in a library reduces the amount of LF that is produced when exotoxin is induced by R-media. All libraries were first normalized by the total protein in the lane. The amount of LF was determined relative to the 100% LF^+^ library. Western blotting was performed two independent times. Columns represent the median and bars represent the range. **B**) Decreasing the amount of LF-producing clones in a library led to reductions in macrophage cytotoxicity. RAW264.7 cells were plated in 96 well plates and treated with bacterial supernatants from exotoxin inducing R media. LDH release was measured and the %LDH release was calculated relative to the 100% LF^+^ supernatant. Experiments were repeated three independent times with at least 4 replicates per run. Asterisks represent a significant difference (One-way ANOVA with Tukey's multiple comparisons test. ****P-value <0.0001). Error bars represent the 95% confidence interval. **C**) Kaplan-Meier curve of mice infected with 2×10^5^ CFU spores subcutaneously in the ear pinna. Mice were observed for 7 days. Solid circles represent mice infected with a library where all clones are LF^+^ (n = 22), open circles represent 40% LF^+^ library (n = 14), empty upward triangles represent 10% LF^+^ library (n = 39), empty downward triangles represent 0.3% LF^+^ clones library (n = 8). Asterisk represents a significant difference from the 100% LF^+^ library (Log rank test, *P-value <0.05).

To test if reducing the proportion of LF^+^ bacilli in a population reduced LF production, the four signature tag libraries were incubated in R media for 6 hours to induce exotoxin production, then the relative amounts of LF and their ability to lyse RAW264.7 cells were analyzed. Quantitative western blotting found there was a reduction in the amount of LF produced by the libraries when induced in R media for 6 hours (Fig. 1A). To determine the consequence of this decrease *in vitro*, the supernatants were added to RAW264.7 cells and the amount of cell death was quantified. RAW264.7 cells are highly sensitive to a rapid, pro-inflammatory death when treated with LT. Therefore, if less LF is being produced by the population when there are fewer LF^+^ bacilli, the ability to lyse macrophages should decrease. LDH release was highest when cells were treated with the LF^+^ strain and slightly lower for the 40% LF^+^ inoculum. The 10% and 0.3% LF^+^ inoculum caused very little LDH release, suggesting that these populations produced a lower level of LF than the 100% LF^+^ and 40% LF^+^ populations (Fig. 1B). To confirm that this decrease in LDH release is not due to poor exotoxin induction, recombinant LF was added back at a concentration of 2.5 µg/mL (27.8 nM) per well and the LDH release was measured. When LF was added back to the 10 and 0.3% LF^+^ supernatants, but not R media alone, there was an increase in LDH release. This suggests that the bacteria still produce sufficient levels of PA but have reduction in LF.

Mice were infected subcutaneously to determine if a decrease in LF-producing bacteria increased survival. These mice were then compared to mice infected with a similar sized inoculum of the 100% LF^+^ library. In agreement with previous publications the infections initiated in the pinna of the ear disseminated to the draining cervical lymph node (cLN), then disseminated throughout the mouse (data not shown) [24], [41]. After dissemination from the cLN, the kidneys were the first organ to luminesce and were used to indicate a disseminated infection. Mice were considered to have a fatal infection when the bacteria had disseminated from the cLN to the kidney. When mice were challenged with the 100% LF^+^ library, approximately 70% of mice had a fatal infection by day 7. When mice were challenged with the 40% LF^+^ library, there was not a significant change in survival (Fig. 1C). Mice that were infected with either the 10% LF^+^ or the 0.3% LF^+^ library, however, had statistically significant increased survival relative to the 100% LF^+^ library (Log rank test, *P*-value  = 0.02). The 10% LF^+^ library was still capable of disseminating in the mouse; however, the 0.3% LF^+^ library was unable to cause any death in mice at a similar sized inoculum. This suggests that decreasing the proportion of LF^+^ bacilli in a population decreases the virulence of the library.

### The 0.3% LF^+^ library cannot colonize the host, but dissemination kinetics and bacterial burdens do not differ between the other libraries

**Figure 2:**
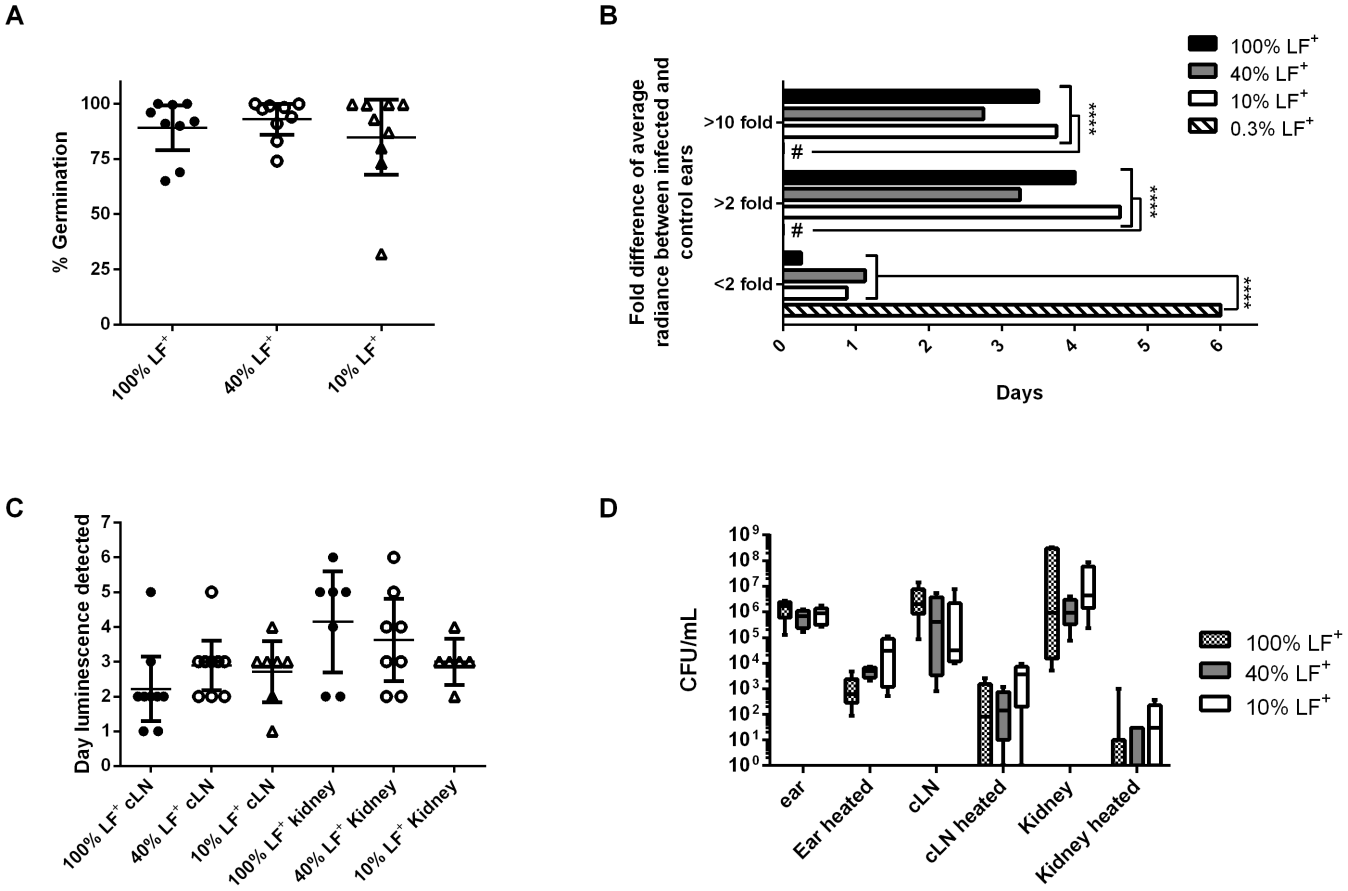
Increased survival was due to defects in colonization or dissemination. **A**) At least 90% of spores in the inoculum germinated 24 hours post infection of ear pinnas. Mice were infected with between 1.5×10^5^–2.0×10^5^ spores of the various libraries and ears were dissected 24 hours post infection. The percentage of germination was measured by comparing the amount of CFU from unheated and heated tissue homogenates. These experiments were done with 3 mice per library and repeated twice. Each symbol represents an individual mouse. Bars represent mean and error bars represent the 95% confidence interval. No statistical difference was found between the populations after performing a one-way ANOVA with Tukey's Multiple Comparison test (*P*-value >0.05). **B**) The number of days luminescence was detected at a particular fold difference over background for each signature tag library was not significantly different. Fold difference represents the increase of the infected ear over the non-infected ear. Solid black bars represent mice infected with the 100% LF^+^ (n = 4), grey bars represent 40% LF^+^ library (n = 8), white bars represent 10% LF^+^ library (n = 8), diagonal hashed bars represent 0.3% LF^+^ library (n = 8). Number signs signify that library never had luminescence detected over background at the given fold difference. Asterisks represent a statistical difference from the 0.3% LF^+^ library (Two way ANOVA with Dunnett's Multiple comparison test; **** *P*-value <0.0001). **C**) There is no temporal difference in dissemination between the signature tag libraries. Columns indicate the average day luminescence was detected in the draining cervical lymph node and kidney with the 100% LF^+^ library (solid circles, n = 9 for cLN, n = 7 for kidney), 40% LF^+^ library (open circles, n = 9 for cLN, n = 8 for kidney), 10% LF^+^ library (upward facing open triangles, n = 7 for cLN, n = 6 for kidney). Mice were observed at least once per day. Error bars represent the standard deviation. No significant difference when comparing the days for either the cLNs or kidneys (One way ANOVA, *P*-value>0.05.) **D**) CFU enumeration (Total bacteria and heat-resistant) in organs infected with differing levels of LF. CFU were enumerated from mice infected with 2×10^5^ CFU spores of either the 100% LF^+^ library (checked boxes, n = 4), 40% LF^+^ signature tag library (gray boxes, n = 6), or 10% LF^+^ signature tag library (white box n = 8). Mice were dissected after luminescence was detected in the kidney, but before mice were moribund. Error bars represent the 10–90 percentiles.

Infection with the 10% and 0.3% LF^+^ library resulted in increased host survival, but it is not known whether this increased survival is due to a defect in colonization, dissemination, or bacterial burden. Previous research has indicated that germination is not dependent on exotoxin production and occurs rapidly after subcutaneous injection in the ear pinna [42], [43]. The luminescent strains of *B. anthracis* used in these studies only produced light when they were metabolically active vegetative bacilli and in exotoxin inducing conditions [24]. To confirm this, mice were subcutaneously infected with the 100% LF^+^, 40% LF^+^, and 10% LF^+^ libraries and the ears were dissected 24 hours post infection. Then the bacterial burdens were analyzed to determine the amount of germination that occurred within 24 hours by comparing CFU from heated and unheated tissue homogenates. Spores are resistant to heat, whereas vegetative bacteria are killed by heat treatment; therefore the heat resistant CFU represent the number of spores in a sample. The median percent germination for all libraries was at least 90% of the population as previously reported (Fig. 2A) [42]. This strongly suggests that germination occurs rapidly in the ear. Replication within the ear could be assessed by measuring the amount of luminescence in the ear during the course of the infection. The 100% LF^+^, 40% LF^+^ and the 10% LF^+^ libraries were capable of replication in the ear, as the bioluminescent radiance in the infected ear would be >10-fold over background for an average of 3.5, 2.8 and 3.8 days, respectively, during the infection period (Fig. 2B). This corresponded with average bacterial burden of 1.5×10^6^ CFU/ear for the 100% LF^+^ library, 6.8×10^5^ CFU/ear for the 40% LF^+^ library, and 9.0×10^5^ CFU/ear for the 10% LF^+^ library when dissemination was detected in these mice. In contrast, the 0.3% LF^+^ library never exceeded >2-fold over background. Further, when the mice were dissected seven days post infection, there was an average of 5.6×10^3^ CFU/ear. This represents a 17-fold decrease from the initial inoculum injected into the ears and 267-fold decrease from the bacterial burdens present when the 100% LF^+^ library infected mice were dissected. This suggests that although the 10% LF^+^ library cannot disseminate as often as the 40% LF^+^ or the 100% LF^+^ library, the 10% LF^+^ library can still replicate within the ear for several days. The 0.3% ΔLF library, however, was not able to colonize the ear.

Since LF is known to be important for colonization and survival in the Sterne-A/J mouse model, the dissemination timing was analyzed in mice with the 40% LF^+^ and 10% LF^+^ libraries and compared to the 100% LF^+^ inoculum. Mice were compared by the day luminescence was first detected in the draining lymph nodes and kidneys. The average time to detection of luminescence in the draining lymph nodes for the 100% LF^+^ library was 2.2 days (95% CI  = 1.3–3.1 days). The 40% and 10% LF^+^ libraries were not significantly different from the 100% LF^+^ library; the average day luminescence was detected in the cLN for the 40% LF^+^ library was 3.0 days (95% CI  = 1.8–4.2 days) and for the 10% LF^+^ library was 2.7 days(95% CI  = 1.8–3.6 days) (Fig. 2C). Similarly, there was no difference between the day of first detection of luminescence in the kidneys of mice. The average day that luminescence was detected in the kidney in mice infected with the 100% LF^+^ library was 4.1 days (95% CI  = 2.8–5.5 days), which was not significantly different from the 40% LF^+^ library average, 3.8 days (95% CI  = 1.8–5.8 days), or the 10% LF^+^ library average, 3.0 days (95% CI  = 2.3–3.7 days). The 0.3% LF^+^ library had no detectable luminescence in the ear, or any other part of the host, during the 7 days the mice were observed.

Lastly, the bacterial burdens in organs were compared when the 100% LF^+^, 40% LF^+^, and 10% LF^+^ libraries disseminated in mice. Since mice with the 0.3% LF^+^ library did not disseminate they were precluded from these analyses. *B. anthracis* infections are asynchronous and therefore mice were compared only if the mice were at the same stage of infection; *i.e*. when luminescence was detected in the kidneys, but before mice were moribund. Though there was variability in the bacterial burdens in tissue, no significant difference could be detected in disseminated organs between the libraries (Fig. 2D). Therefore, we conclude that if *B. anthracis* can disseminate systemically that no significant differences occur in the bacterial burden.

### ΔLF clones survive and disseminate throughout the host when there is a 60% reduction of LF-producing bacteria

Since there were increases in host survival with the 10% LF^+^ library, but no defects in dissemination kinetics or bacterial burden, whether this observation reflected the failure of ΔLF clones to replicate or disseminate in the host was tested. Clonal analysis was used to determine if the ΔLF clones could survive *in vivo* and determine how well LF can be complemented *in trans* by the LF producing bacilli for the 40% LF^+^ and 10% LF^+^ libraries. Clonal analysis uses a library of clones that are differentiated only by a single tag or marker. Animal models can be infected with these libraries and then dissected to determine the signature tag identities present in the host. The changes of the proportions of tagged bacteria during dissemination through the host can then be used to determine how the bacterial population was altered during infection. Previous research, however, has demonstrated that *in vivo* bottlenecks occur in *B. anthracis* infections in mice [33], [44]. A bottleneck is a decrease in population size that leads to a reduction in the genetic diversity of said population. A consequence of these bottlenecks is that higher rates of genetic drift occur for the population [45]. However in pathogenesis research, bottlenecks have been investigated in order to identify a moment in infection where the host imposed a barrier to the pathogen's dissemination [33], [46]. While stringent bottlenecks could allow for the identification of sites where LF is required for survival and dissemination, it can also complicate analysis of *in trans* complementation in our model. Clonal analysis was first performed with the 100% LF^+^ library to determine if a stringent bottleneck occurred in the subcutaneous model. Mice were infected with between 2.0×10^5^ and 5.0×10^5^ spore CFU in the pinna of the ear. The infection was allowed to disseminate until light was detected in the kidney, indicating that the host is bacteremic.

**Figure 3:**
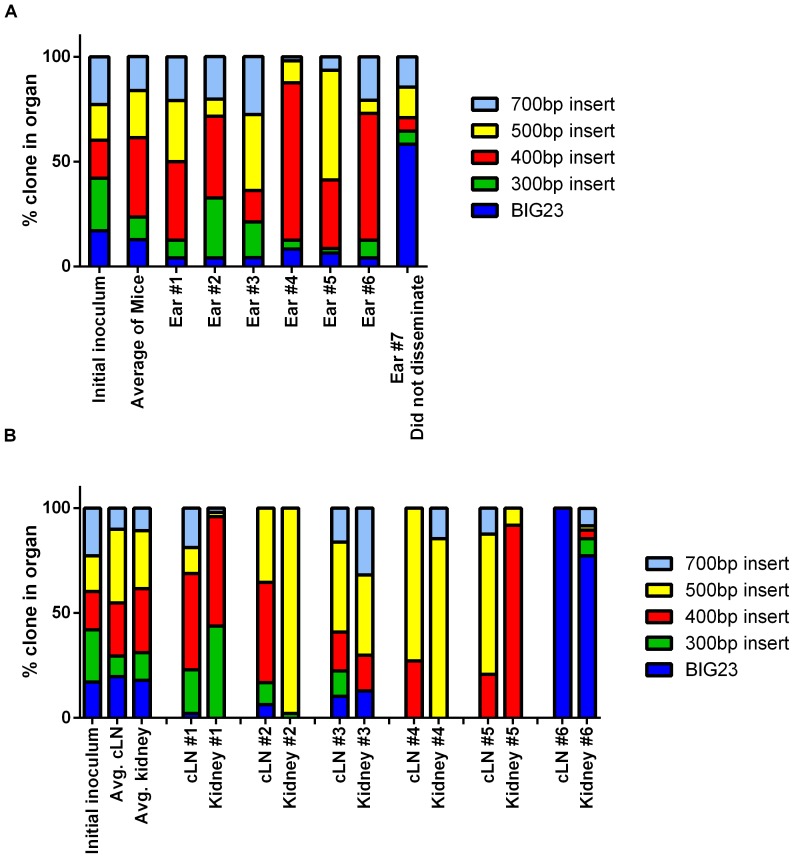
Multiple bottlenecks occur in the subcutaneous routes, but several clones are capable of dissemination. **A**) Graphs indicate the signature tag proportions found of the bacterial population that resided in the ear of mice infected with the 100% LF^+^ library. Each column is a stacked percentage bar where each clone is represented by a different color. **B**) Bottlenecks often occur in the draining cLN and are always present by the time the bacteria reach the kidneys. Graphs indicate the signature tag proportions found of the bacterial population that resided in the cLN or kidney of mice infected with the 100% LF^+^ library. Each column is a stacked percentage bar where each clone is represented by a different color.

When signature tag proportions were analyzed in the ear infected with the 100% LF^+^ library, four of seven mice had at least two clones comprise >70% of the population, suggesting a bottleneck occurred in the ears of these mice (Fig. 3A). Mouse #7's bacterial population did not subsequently disseminate, but a bottleneck still occurred. The other three mice did not indicate a bottleneck happened in the ear since no clone comprised more than 45% of the population. This suggests that a bottleneck can occur in the ear of mice, but does not necessarily occur in every infection. As a control for *in vivo* selection against the DNA tag interrupting the *eag* locus, the signature tag proportions found in the ears were compared to the initial inoculum. If there is no *in vivo* selection in an organ, the signature tag proportions should be statistically equal to the inoculum. When these proportions were averaged for all 6 mice there was not a significant difference between the average proportions and the initial inoculum (Fig. 3A, Fisher exact test *P-*value  = 0.132). This suggests that an *in vivo* growth defect does not occur from a tag interrupting the *eag* locus when all clones produce LF. When the cLNs were analyzed, four out of six mice had a bottleneck given the above criteria (70% of the population comprised of ≤2 clones). The three mice with bottlenecks in the ear corresponded with three of the cLNs with bottlenecks. The averaged proportion of the clones did not significantly vary from the inoculum, implying that all clones were equally capable of reaching the cLN (Fig. 3B, Fisher's exact test *P*-value  = 0.063). Therefore, a bottleneck can occur in either the ear or as the bacteria migrate from the ear to the cLN. When the disseminated populations in the kidneys were examined, there were pronounced bottlenecks in 7 of 8 mice with 1 or 2 clones comprising >85% of the population (Fig 3B). Similar to the bacterial populations in the cLN, the averaged kidney populations did not have a significant difference from the initial inoculum, suggesting that the bottlenecks were not due to host selection against specific tagged clones (Fisher's exact test *P*-value  = 0.14). Those mice that did not have a bottleneck in the lymph node passed through a bottleneck en route to the kidney. Thus, the subcutaneous route has multiple bottlenecks that occur, with the most stringent typically occurring between the cLN and the kidney. Yet, the subcutaneous route demonstrated that several clones are present in the ears, cLNs, and at times the kidney. Disseminated populations of bacteria from intranasal routes, however, bottleneck either at the initial site of infection or as the bacteria disseminate to the lymphatics [33]. This early bottleneck would lessen or preclude the ability of the LF^+^ clones to complement the ΔLF clones during dissemination. This would greatly increase the number of animals needed to determine if the ΔLF clones could still disseminate. Therefore, the subcutaneous model of infection is the most appropriate model for testing the role of LF by *in trans* complementation.

**Figure 4:**
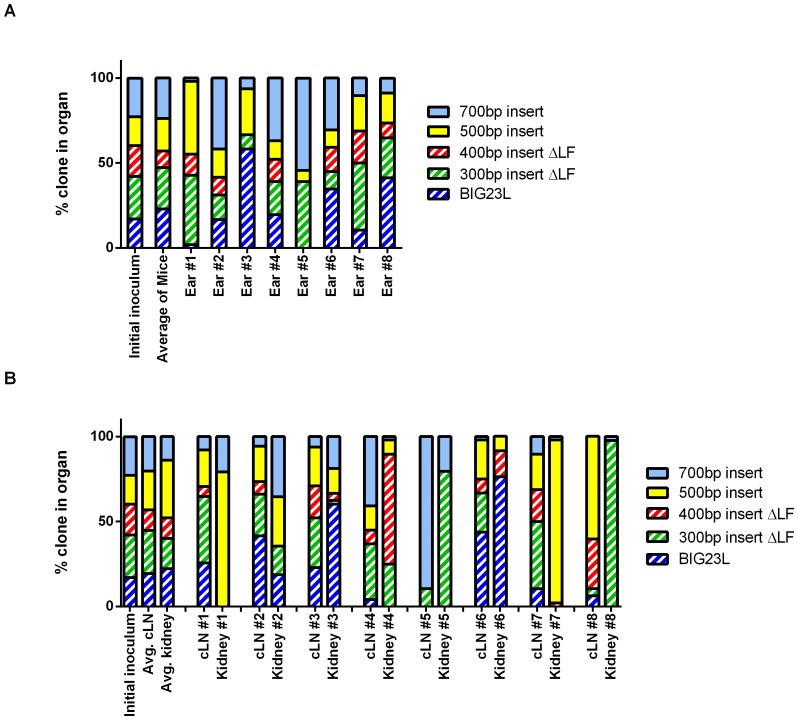
ΔLF clones can be trans complemented when 40% of the library produces LF. **A**) ΔLF clones are capable of colonization and replication when LF is reduced by 60%. The signature tag proportions found of the bacterial population that resided in the ear of mice infected with the 40% LF^+^ library. Each column is a stacked percentage bar where each clone is represented by a different color. **B**) ΔLF clones disseminate to the cLN and kidneys when there is a 60% reduction in LF-producing bacteria. The signature tag proportions found of the bacterial population that resided in the cLN and kidney of mice infected with the 40% LF^+^ library. Each column is a stacked percentage bar where each clone is represented by a different color.

When the signature tag proportions were analyzed in the ears of mice that received the 40% LF^+^ library, the signature tag proportions were not statistically different from the initial inoculum (Fig. 4A, Fisher's Exact Test *P*-value  = 0.81). Similar to the 100% LF^+^ inoculum, half of the mice had >70% of the population be comprised of ≤2 clones. The draining cLNs from mice infected with the 40% LF^+^ library only had a bottleneck in 1 of the 8 mice; the other mice had no sign of a bottleneck in the cLN (Fig. 4B). When averaged, the signature tag proportions in the cLNs did not significantly differ from the initial inoculum, suggesting that all clones were equally capable of reaching and replicating within the draining lymphatics (Fig. 4B, Fisher's Exact Test *P*-value  = 0.89). Lastly, the kidneys were analyzed to determine if the ΔLF clones could disseminate throughout the host. Similar to the 100% LF^+^ library, the signature tag proportions suggested a stringent bottleneck occurred between the cLN and the kidneys. Five of the eight mice (62.5%) had a majority of the population composed of ΔLF clones and there was no statistical difference between the averaged signature tag proportions in the kidneys and the initial inoculum (Fig. 4B, Fisher's Exact Test *P*-value  = 0.29). These data suggest that the LF^+^ clones are capable of complementing *in trans* for the ΔLF clones to such an extent as to permit the dissemination of the normally avirulent ΔLF throughout the mouse.

### The ΔLF clones have decreased proportions and fewer clones disseminate when only 10% of the population contains LF-producing bacteria

**Figure 5:**
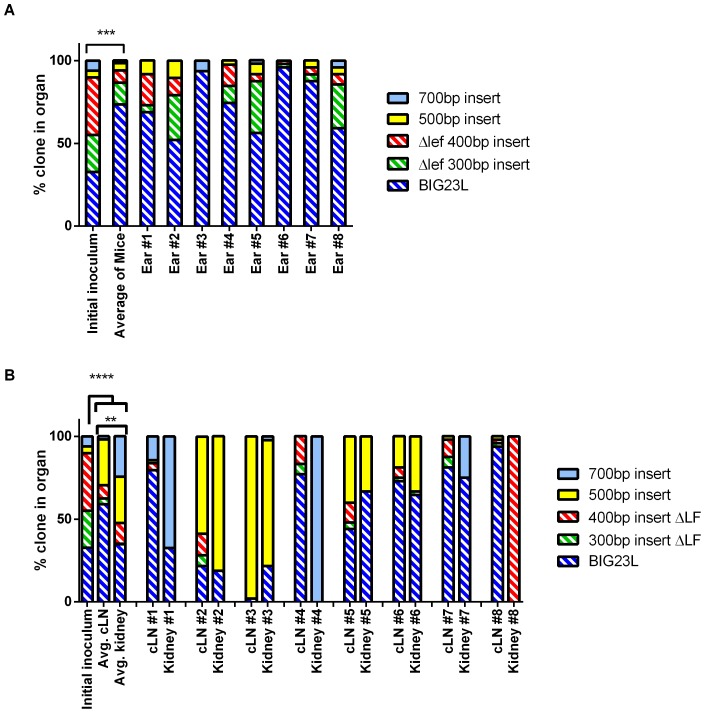
ΔLF clones are less capable of survival and dissemination when 10% of the library produces LF. **A**) BIG23L is the dominant clone in the ear of mice when there is a 90% reduction of LF-producing bacteria in the inoculum. The signature tag proportions found in the bacterial population that resided in the ear of mice infected with the 10% LF^+^ library. Each column is a stacked percentage bar where each clone is represented by a different color. Asterisk represents a significant difference from the initial inoculum (Fisher's Exact test, *** *P*-value <0.001) **B**) ΔLF clones are less abundant in the disseminated population when LF is reduced by 90%. The signature tag proportions found of the bacterial population that resided in the cLN and kidneys of mice infected with the 10% LF^+^ library. Each column is a stacked percentage bar where each clone is represented by a different color. Asterisk represents a significant difference from the initial inoculum (Fisher's Exact test, ** *P*-value <0.01; ****P-value <0.0001).

Mice infected with the 10% LF^+^ clonal library were analyzed to determine if the ΔLF clones could survive and replicate as they disseminate through the host in the presence of limited LF production. In mice where the infection disseminated through the host, the ears had an increased proportion of BIG23L relative to the initial inoculum in all eight mice (Fig. 5A). This increase in BIG23L came at the expense of the other ΔLF strains as on average 93% of the population was ΔLF and resulted in a significant difference from the initial inoculum delivered (Fig. 5A, Fisher's exact test *P-*value  = 0.00017). This result was surprising, as there was no indication that the 300 bp or 400 bp insert clones having any *in vivo* survival defects when the mice had a 40% LF^+^ or 100% LF^+^ in the ear (Fig 3A and 4A). Moreover, there were no growth or *in vivo* dissemination defects for the 300 bp or 400 bp insert in inhalational infections [33]. After disseminating from the ear to the cLN, LF^+^ clones comprised a significantly greater percentage of the population, including 2 mice with a majority of the population comprised by a LF^+^ clone (Fig. 5B). The average proportions of clones in the cLN demonstrate that there is a decrease in ΔLF clones and in particular the BIG23L clone (Fig. 5B). The decrease in ΔLF clones relative to the ear continued when the kidneys were analyzed. In half of the kidneys analyzed by clonal analysis, the LF^+^ clones grew to represent the majority of the population. Further, 7 of the 8 mice had the percentage of LF^+^ clones at least double from their proportion in the ear (Fig. 5B). This suggests that the LF^+^ clones are more likely to disseminate when 90% of the population cannot produce LF.

**Figure 6:**
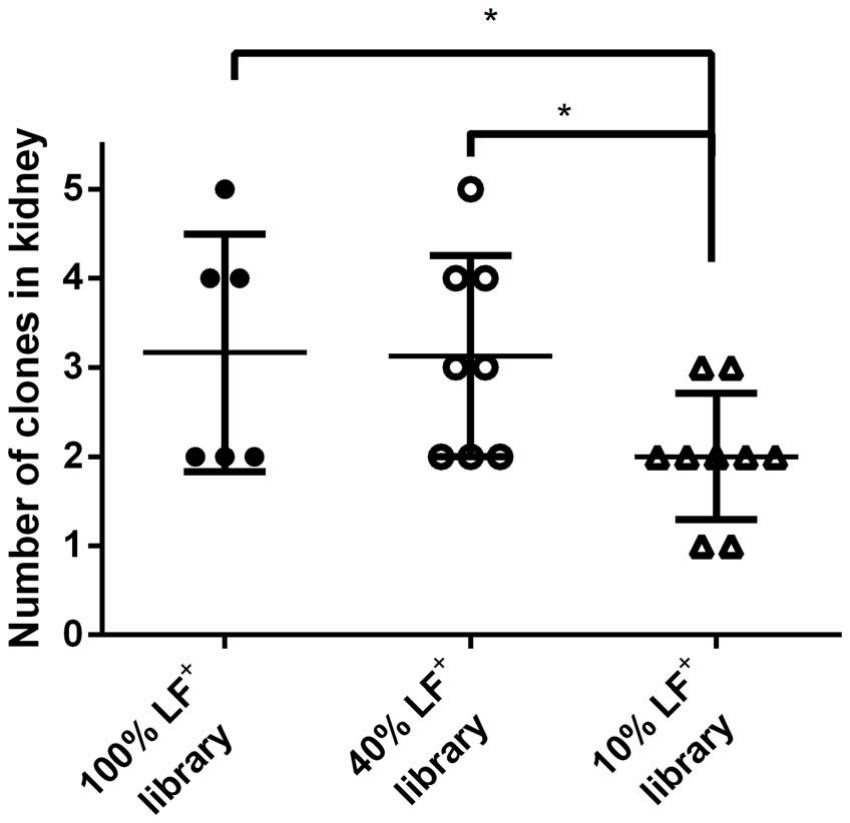
Fewer clones disseminate when the library only has 10% of the population produce LF. There are fewer clones that disseminate when LF-producing bacteria are reduced by 90% in the inoculum. The number of clones that disseminated to the kidneys were enumerated and compared between the libraries. Each symbol represents a mouse. Bars represent the mean and error bars represent the standard deviation. Asterisks represent a statistical difference between the means (Student's T Test, *P-value  = 0.044).

Lastly, the number of clones that were able to disseminate to the kidney were compared between the 100% LF^+^, 40% LF^+^ and 10% LF^+^ library. There was a significant decrease in the amount of clones that were able to disseminate to the kidneys in the 10% LF^+^ library compared to either the 100% LF^+^ or 40% LF^+^ library (Fig. 6). This suggests that LF influences the ability of clones to pass through the bottlenecks in a subcutaneous infection.

## Discussion

The production of LF *in vivo* is an important virulence factor that promotes bacterial survival and contributes to host mortality. While genetic deletions of LF have highlighted its importance for disease, the relative amounts of LF needed in the early phases of pathogenesis are poorly understood. A principal finding of this study was that decreasing amounts of LF^+^ bacilli in the inoculum had discrete effects on the local site of colonization. When the population was 40% LF^+^ bacteria, there were no differences in survival, colonization, or bacterial burden in the analyzed tissues. A further reduction of LF^+^ bacilli to 10% of the population led to an increase in host survival. Interestingly, on those occasions when dissemination did occur with the 10% LF^+^ population, there was no significant change in dissemination kinetics, bacterial burden, and colonization of the ears compared to the 40% or 100% LF^+^ population. This suggests that the amount of LF produced by the 10% LF^+^ library is sufficient for colonization, but resulted in less frequent dissemination. The library with the lowest amount of LF^+^ bacilli, the 0.3% LF^+^ library, never had luminescence that was detectable over background (≥2-fold), implying the bacteria colonized the ear poorly. As such, it appears that when the LF^+^ clones are reduced by 90%, the population is less likely to disseminate from the ear. However, the inoculum must have more than 0.3% of the population producing LF in order to colonize the ear. These data suggest that LF first allows for survival within the portal of entry after germination. This primary role allows bacilli to continually produce the exotoxin and multiply until a threshold is reached which allows for dissemination from the initial site. Thus LF plays two roles in infection: a primary role for colonization and a secondary role for dissemination.

Anthrax is an acute disease in which the bacteria rapidly disseminate through the host [22]–[24], [33]. Given this acute nature, a promising area for therapeutic development would be the early stages when the bacteria begin to overwhelm the host defenses in order to disseminate. When the amount of LF-producing strains was reduced to 10% of the population, the host survival increased. Yet, there were no significant differences between the bacterial burden, dissemination kinetics, or ability to colonize the host. This suggests that in a murine subcutaneous infection, the initial site of infection is a crucial point that determines whether the host will survive the infection. Similar studies have found that removing the infected portion of the ear or genetically deleting the CMG2 from myeloid cells leads to increases in mouse survival [26], [36]. Our findings, however, imply that LF is critical for this survival and dissemination from the initial site.

Reducing the number of LF producing bacilli illustrates the protective effect of the exotoxin on a population of bacteria. Our current and previous findings found no difference in ability to disseminate between strains with a DNA tag interrupted the *eag* gene and its parental counterpart when there was at least 40% of the population produced LF. Furthermore, when *eag* was interrupted with the DNA tags, there was no difference in the *in vitro* growth rates. Yet, in populations where 90% of the population was ΔLF, there was an increase in the *eag* ^+^ BIG23L clone. This change predominantly came due to a decrease in the 300 bp insert and 400 bp insert ΔLF clones, since average BIG23L proportion of the population doubled while the LF^+^ population only decreased from 10% to 7% of the population. While EA1, the protein encoded by *eag*, does not seem to have a direct role in pathogenesis, it is a major component of the S-layer. The S-layer has several proteins that are attached to it which have been shown to be important for adhesion, pathogenesis, and iron uptake [47]–[49]. It is possible that in this animal model the decrease in LF producing strains reveals that the S-layer may play a role in pathogenesis, but the exotoxin obscures the defect in most circumstances. We speculate that the exotoxin can mask minor survival defects *in vivo* by effectively inhibiting the host response. Since bacterial burdens are the same after dissemination despite increases in host survival, clonal analysis may be a more sensitive method to measure decreased virulence.

In the inhalational rabbit model of anthrax, ΔPA strains cannot be *trans* complemented when co-infected with the fully toxigenic strain [22]. As such, it was surprising to find that both the 40% LF^+^ and 10% LF^+^ populations had the LF^+^ clones *trans* complement the ΔLF clones. Since this study uses different animal models, bacterial strains, and infection routes, direct comparisons between these data are difficult. Further, since capsulated non-toxigenic bacilli disseminate in mice, the strains used in Lovchik *et al*.'s rabbit model would be inappropriate for the analysis of the role of LF early in infection. Nonetheless, mouse models have several advantages over the rabbit model which can be used to answer questions regarding early *B. anthracis* pathogenesis. Notably, mice permit the use of bioluminescent imaging, which allowed for the observation that the increased host survival was due to the decreased dissemination in the 10% LF^+^ library and the inability of the 0.3% LF^+^ library to colonize the ear. Further, the use of mice infected with bioluminescent *B. anthracis* indicated that the average time of dissemination from the ear to the draining lymph node and from the lymph node to the kidney are statistically indistinguishable despite increased host survival. This was crucial for determining that early events in *B. anthracis* infections either lead to clearance and survival or dissemination and death.

LF, and the exotoxin in general, may influence the number of signature-tagged clones that disseminate from the initial site of infection. Previous research has noted that the host tissue environment where the infection establishes impacts the population structure as it disseminates [33]. In this study clonal analysis indicated that when LF is reduced to the level produced by the 10% LF^+^ inoculum fewer clones comprise the disseminated population. Given that different routes allow distinct numbers of clones to disseminate, this may also explain the discrepancy between our results and Lovchik *et al* [22]. It is quite possible LF acts in close proximity to the secreting bacilli when being transported to the lymph nodes intracellularly, but is less influential in other routes of infection. This suggests that LF, and the exotoxin as a whole, plays a role in defining the stringency of the population bottleneck.

While the exact cause of these subcutaneous bottlenecks is unknown, we can speculate as to why they occur. Subcutaneous infections rapidly germinate and encounter innate immune cells in the early stages of infections [42]. The bottlenecks in the ear may be from inoculums that were not able to produce enough exotoxin in order to limit or prevent the initial immune response. Thus, we speculate that the ear bottleneck is due to the initial immune response that fails to clear the bacteria. We favor the following two scenarios to describe the bottleneck that was detected in or after disseminating from the draining lymph node. In the first scenario, the bottleneck could occur due to the first or first few bacteria that colonize the draining lymph node. This migration-based bottleneck has been suggested before for *B. anthracis* [33], [44]. A second scenario could be that exotoxin has yet to inactivate the immune cells within the draining lymph node. However, previous findings have noted increased LF levels in the draining cervical lymph node before bacteria had disseminated from the ear [27]. Given the high levels of LF that have been reported when bacteria are in the draining lymph node, it seems most likely that the bottleneck occurs as the first few bacteria migrate to the bloodstream.

*B. anthracis* dissemination has two widely accepted models of dissemination through the host: the Trojan horse model and the Jailbreak model [36], [50]. The Trojan horse model involves spores being internalized by professional phagocytes which deliver the spores to the lymph nodes. Once in the lymph nodes, the spores germinate, escape the phagocyte, and begin the infection in the draining lymph node [50]. In contrast to the Trojan horse model of infection, the Jailbreak model argues that germination occurs extracellularly and the exotoxin and/or secreted proteases lead to the destruction of the tissue barriers and disrupt the host immune response [36]. The subcutaneous mouse model appears to support the Jailbreak model as spores can germinate extracellularly in the ear and debridement of infected ear tissue increases host survival [41], [42]. This could explain how population-based decreases in LF led to an overall decrease in dissemination for the ear as decreased levels of LF-producing bacteria would be less likely to suppress the immune response or breakdown tissue barrier function. Yet, a bottleneck occurred during the disseminated infections described here and only a few bacteria were capable of causing an infection which led to the host death. This argues that exotoxin production from all bacteria is acting synergistically to increase the chance of dissemination and host death while at the same time any single bacterium can disseminate and cause the death of the animal. This resembles the partial synergistic action hypothesis in infection [51]. It would be interesting to see if this infection action model is applicable to other pathogens or routes of *B. anthracis* infection.

The route of infection or which specific exotoxin component that is being complemented may also play a role in the ability of *in trans* complementation to occur. Some animal models were found to have a high bacterial burden in the lung associated lymph nodes but not in the lungs, suggesting that the spores were trafficked to the lung associated lymph nodes by phagocytes acting as a Trojan horse [22]. In contrast to these inhalational infections, subcutaneous ear infections are known to spread from the colonized tissue in the ear, rather than from spores that migrated to the lymph node [36]. This raises the interesting possibility that the amount of LF needed in a site is dependent on the environment that is colonized. Another possibility for the differences from others is that different exotoxin components were being complemented [22]. It is possible that while LF is able to be complemented, PA cannot; perhaps reflecting unappreciated differences between these two exotoxin component's biochemical functions, such as the timing of formation of biologically active holotoxin relative to the proximity to the bacterium. Further research is necessary to understand how exotoxin components interact *in vivo* as well as whether these interactions are similar in all tissues.

While much has been gained in previous analysis of the anthrax exotoxin in disease and dissemination there are still areas in further need of study. Our research indicates that a certain amount of LF^+^ strains in a population are necessary for dissemination in a subcutaneous model of infection, but a lower level is needed for the initial colonization. Thus, it follows that a greater focus should be placed on understanding the relative concentrations of LF that exist in the host in particular time points in the infection. Such advances in basic science would likely hold great promise in development and validation of future therapeutics in animal models.
